# Assessing corneal dendritic cells in glucose dysregulation small‐fibre neuropathy

**DOI:** 10.1111/jns.12671

**Published:** 2024-11-12

**Authors:** Juan Francisco Idiaquez, Carolina Barnett‐Tapia, Bruce A. Perkins, Vera Bril

**Affiliations:** ^1^ Ellen and Martin Prosserman Centre for Neuromuscular Diseases, Division of Neurology, Department of Medicine University Health Network, University of Toronto Toronto Canada; ^2^ Division of Endocrinology and Metabolism University of Toronto, and the Leadership Sinai Centre for Diabetes, Sinai Health Toronto Canada

**Keywords:** corneal confocal microscopy, corneal dendritic cells, impaired glucose tolerance, small‐fibre neuropathy

## Abstract

**Background and Aims:**

Small‐fibre neuropathy (SFN) is associated with glucose dysregulation, including impaired glucose tolerance (IGT) and type 2 diabetes (T2D). Corneal confocal microscopy (CCM) offers a non‐invasive tool to assess corneal nerve damage and dendritic cell density (DCD). In this study, we investigated corneal DCD in patients with SFN and glucose dysregulation, defined as IGT or T2D.

**Methods:**

We enrolled 38 patients with SFN + glucose dysregulation, 51 with SFN + non‐glucose dysregulation and 20 healthy controls. All participants underwent neurological examination, neurophysiology and CCM.

**Results:**

Individuals with SFN and glucose dysregulation had higher DCD compared with healthy controls (*p* = .01), and mature DCD was higher in IGT SFN patients than in T2D patients.

**Interpretation:**

Higher DCD in IGT compared with controls and patients with established T2D may suggest that DCD is a biomarker of early neuropathy.

## 1 INTRODUCTION

Impaired glucose tolerance (IGT) represents an early stage of glucose dysregulation and is associated with small‐fibre neuropathy (SFN).^1^, ^2^ SFN can also manifest in patients with recently diagnosed type 2 diabetes,^3^ often preceding diabetic complications. Corneal confocal microscopy (CCM) is a high‐resolution imaging technique that is particularly useful in diagnosing SFN by measuring corneal nerve fibre length (CNFL) and also allows for the quantification of dendritic cell density (DCD).^4^ CCM is a non‐invasive and rapid technique that provides structural insights into nerve fibre loss, with diagnostic properties comparable to intraepidermal nerve fibre density assessments.^5^, ^6^

Chronic hyperglycaemia negatively impacts corneal nerve function and epithelial cell repair.^7^ The role and pathogenesis of DC accumulation in diabetic neuropathy are not well elucidated. Both animal and clinical studies have reported increased DCD in subjects with type 1 and type 2 diabetes.^8^, ^9^, ^10^ Increased DCD in diabetic patients, particularly in the earlier phases of corneal nerve damage, suggests an immune‐mediated contribution to corneal nerve damage in diabetes.^10^ Additionally, studies show that DCD correlates with corneal nerve loss in various diabetes types, including type 1, type 2 and latent autoimmune diabetes in adults.^11^

Patients with diabetic SFN and neuropathic pain had increased DCs in the epidermis, potentially aiding nerve repair.^12^, ^13^ A significant reduction in cutaneous DCD has been observed in newly diagnosed type 2 diabetes, potentially contributing to a metabolically associated, local imbalance in immune cells in peripheral nerves and increasing the risk of complications like polyneuropathy and foot ulcers.^14^ Research comparing SFN patients with healthy controls found a significant increase in DCD in SFN patients, suggesting that DCs may serve as inflammatory markers in various forms of small‐fibre polyneuropathy.^15^

Given these findings, the role of DCs in SFN, particularly in the context of glucose dysregulation, is of significant interest. While previous research has established that DCD is higher in SFN patients compared with healthy controls, it remains unclear whether DCD differs between forms of SFN—specifically those associated with glucose dysregulation (IGT and type 2 diabetes) and those occurring without glucose dysregulation. In this study, we aimed to examine and compare corneal DCs in patients with SFN related to IGT and type 2 diabetes, as well as in SFN patients without glucose dysregulation, alongside healthy controls.

## 2 PATIENTS AND METHODS

### 2.1 Study participants

We performed a retrospective cross‐sectional single‐centre study of patients with established SFN who attended the Prosserman Family Neuromuscular Clinic at Toronto General Hospital between 2018 and 2021. The study protocol was approved by the Research Ethics Board of the University Health Network, who waived informed consent.

Participants included individuals aged ≥18 years diagnosed with SFN based on consistent clinical symptoms and/or signs, plus normal nerve conduction studies and two out of three abnormal small nerve fibre tests (CCM, Laser Doppler Imaging flare and Quantitative Thermal Thresholds).^16^ Additionally, patients with documented type 2 diabetes or IGT were included based on medical records and verified using American Diabetes Association criteria for plasma glucose levels, including haemoglobin A1c (HbA1c), fasting plasma glucose, random elevated glucose with symptoms or abnormal 2‐h oral glucose tolerance test.^17^ SFN participants were categorized into those with glucose dysregulation (IGT and type 2 diabetes) and non‐glucose dysregulation groups.

### 2.2 Data collection

Medical records were reviewed to extract demographic and clinical data, including age, gender, medical history, nerve conduction study results, Toronto Clinical Neuropathy Score (TCNS), modified TCNS,^18^, ^19^ Overall Neuropathy Limitation Scale (ONLS)^20^ and Rasch‐built Overall Disability Scale (RODS).^21^ CCM was used to assess SFN. A cohort of 20 healthy controls with normative CCM data from prior study was used for comparison.^22^ SFN patients were categorized into severity groups based on the TCNS score: mild neuropathy (scores 6–8), moderate neuropathy (scores 9–11) and severe neuropathy (scores ≥12).^18^, ^19^

### 2.3 Nerve conduction studies (NCS)

NCS were conducted using the Sierra Wave instrument (Cadwell Laboratories Inc., Kennewick, WA, USA) using age‐ and height‐adjusted reference values from the Toronto General Hospital electrophysiology laboratory.

### 2.4 CCM image acquisition

Corneal examination was performed using the Rostock Cornea Module of the Heidelberg Tomograph II (Heidelberg Engineering, Smithfield, Rhode Island, United States). Patients received topical anaesthetic and tear gel to optimize corneal contact with a disposable sterile cap on the lens. Automated imaging captured 40 contiguous 0.3‐mm^2^ digital pictures per cornea, recorded at 1.3 μm increments to a total depth of 50 μm. This process was repeated twice per eye to ensure precise data acquisition. A trained examiner selected six high‐quality images (three per eye) from the central cornea for CNFL and DCD analysis as part of a quality control protocol. These images were then evaluated in a masked fashion to eliminate bias, and the image with the highest fibre density was chosen for final analysis.

### 2.5 Dendritic cell quantification

**FIGURE 1:**
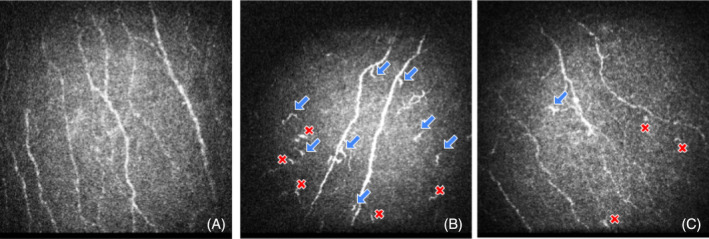
Representative in vivo confocal microscopy images of the central cornea showing highly reflective dendritic cells (DCs). Mature DCs are indicated by blue arrows, and an immature DC by a red “X.” (A) Control subject, (B) patient with SFN related to glucose dysregulation and (C) patient with non‐glucose dysregulation SFN.

ImageJ software (version 1.41, National Institutes of Health, USA) with a cell‐count plugin was used for blinded quantification of CNFL and DCD. The presence or absence of highly reflective cells was assessed. Then, the number of highly reflective cells DC (Figure 1) was categorized as immature (less than 50 μm without dendritic structures) or mature (with dendritic structures).^23^, ^24^ The reliability of these DCD measurements in the corneal sub‐basal epithelium has been confirmed.^23^, ^24^

### 2.6 Statistical analysis

Statistical analysis was performed using R (R Foundation for Statistical Computing version 4.0.4). Descriptive statistics such as mean ± standard deviation or median with interquartile range were used to summarize demographic and clinical characteristics. For the primary analysis, we compared corneal DCD between three groups: SFN with glucose dysregulation, SFN without glucose dysregulation and healthy controls. Analysis of variance (ANOVA) or non‐parametric equivalents were used to compare DCD among these groups. Spearman's rank correlation coefficient was used for correlation analysis. In our cohort of subjects with SFN related to glucose dysregulation, we estimated 80% power to detect a significant difference in DCD between groups. This estimate was based on *α* = .05, assuming an anticipated 70% DCD in the SFN with glucose dysregulation compared with healthy controls, derived from a report of a comparably aged diabetic neuropathy cohort.^10^

## 3 RESULTS

We included 89 individuals with SFN, of which 38 had glucose dysregulation (18 IGT, 20 type 2 diabetes). The non‐glucose dysregulation group had 51 individuals, 43 with idiopathic SFN and 8 with SFN due to other causes: monoclonal gammopathy of undetermined significance (*n* = 2), toxic (*n* = 3), vitamin B12 deficiency (*n* = 2) and human immunodeficiency virus (*n* = 1). We also included 20 controls.

Table 1 presents demographic data and clinical measures, including age, sex distribution, HbA1C levels, and sensory and neuropathic indices (RODS, ONLS, MTCNS, TCNS, CNFL, DCD), comparing individuals with glucose dysregulation SFN (*n* = 38), non‐glucose dysregulation SFN (*n* = 51) and healthy controls (*n* = 20).

**TABLE 1:** Clinical characteristics stratified by glucose dysregulation classification.

Variable (mean (SD))	Glucose dysregulation SFN (*n* = 38)	Non‐glucose dysregulation SFN (*n* = 51)	Healthy control (*n* = 20)	*p* value (GD vs. NGD)	*p* value (GD vs. healthy control)	*p* value (NGD vs. healthy control)	*p* value (ANOVA)
Sex, (Male)	17 (44.74)	21 (41.18)	10 (50%)				
Age (years)	59.45 (10.40)	55.98 (11.82)	41.3 (17.3)	.154			<.56
HbA1C	6.46 (0.90)	5.45 (0.26)	NaN (NA)	**<.001**	‐	‐	**<.001**
RODS	42.26 (7.81)	41.24 (6.73)	NaN (NA)	.567	‐	‐	.567
ONLS	1.16 (1.49)	1.05 (1.08)	NaN (NA)	.732	‐	‐	.732
MTCNS	10.39 (7.25)	12.66 (6.44)	NaN (NA)	.239	‐	‐	.239
TCNS	7.41 (4.03)	7.93 (3.95)	NaN (NA)	.552	‐	‐	.552
CNFL (mm/mm^2^)	5.21 (1.85)	4.58 (1.79)	12.53 (2.87)	.111	**<.001**	**<.001**	.105
DCDM (cells/mm^2^)	10.40 (8.45)	8.11 (8.47)	1.47 (1.90)	.211	**<.001**	**<.001**	**<.001**
DCDI (cells/mm^2^)	12.41 (13.42)	9.65 (11.74)	2.17 (3.35)	.306	**<.001**	.**003**	.**002**
DCDT (cells/mm^2^)	11.40 (9.39)	8.88 (9.00)	1.82 (2.16)	.203	**<.001**	**<.001**	**<.001**

Abbreviations: CNFL, Corneal Nerve Fibre Length; DCDI, Dendritic Cell Density Immature; DCDM, Dendritic Cell Density Mature; DCDT, Dendritic Cell Density Total; GD, Glucose dysregulation; HbA1C, Haemoglobin A1C; LDI, Laser Doppler Imaging; MTCNS, Modified Toronto Clinical Neuropathy Score; NGD, non‐Glucose dysregulation; ONLS, Overall Neuropathy Limitations Scale; RODS, Rasch‐built Overall Disability Scale; TCNS, Toronto Clinical Neuropathy Score.

**FIGURE 2:**
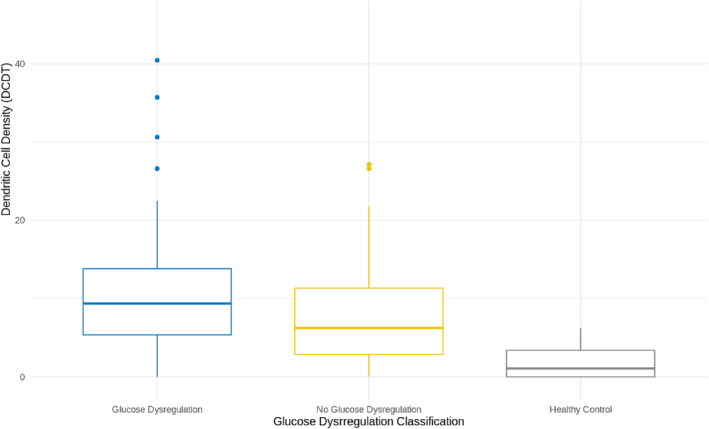
Comparison of dendritic cell density (DCD) (cells/mm^2^) across glucose dysregulation classifications. Boxplots show median and interquartile ranges for subjects with SFN with glucose dysregulation (GD), SFN without glucose dysregulation and healthy controls. Outliers are represented by individual data points.

Both groups—SFN with and without glucose dysregulation—had significantly higher DCD than healthy controls (*p*‐value = <.001). There was no significant difference in DCD between the GD and no GD groups (*p* = .081, Figure 2).

**FIGURE 3:**
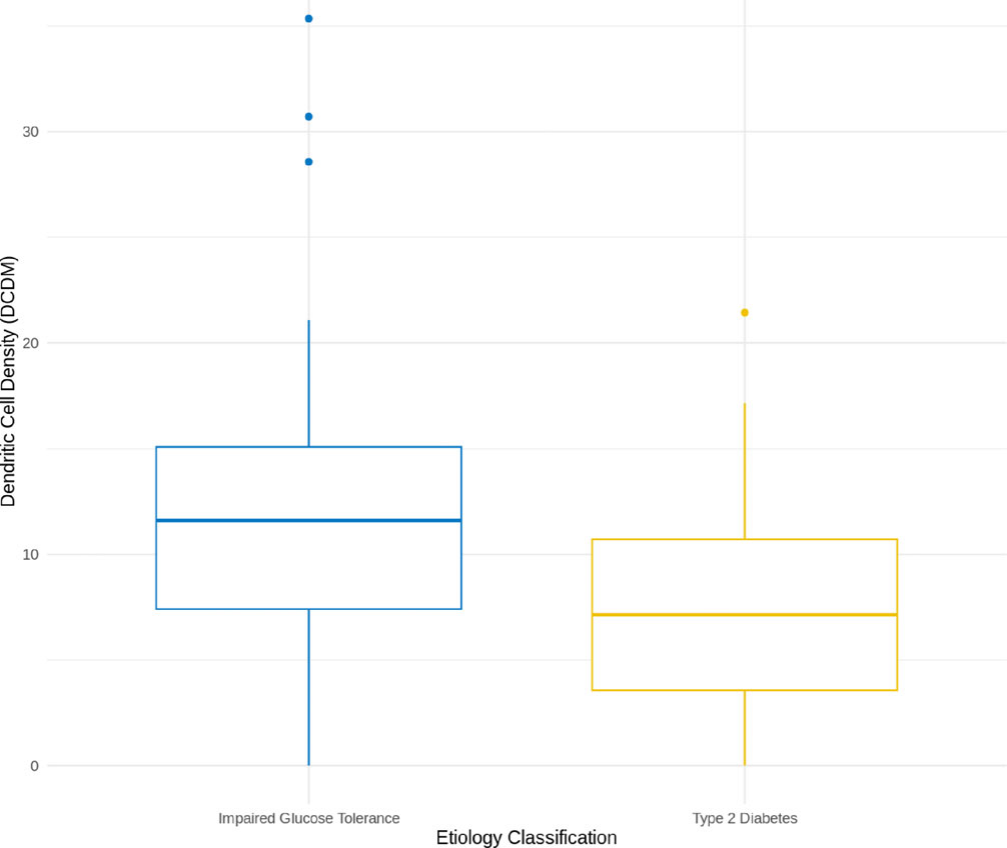
Boxplots comparing dendritic cell density (DCD) (cells/mm^2^) across aetiology groups: IGT and Type 2 Diabetes. DCDM represents the density of mature dendritic cells measured in the study participants. Outliers are represented by individual data points.

Table 2 shows significant differences in HbA1C levels between healthy controls and patients with IGT or type 2 diabetes, while other characteristics like age and sex did not differ significantly between the groups. In particular, individuals with IGT have significantly higher mature DCD levels compared with those with type 2 diabetes (*p*‐value = .045) (Figure 3). HbA1c showed a weak positive correlation with an r coefficient of 0.21, with mature DCD and total DCD (*p*‐values <.0001), but no significant correlation with other variables (RODS, ONLS, TCNS, mTCNS, CNFD, CNFL).

**TABLE 2:** Clinical characteristics stratified by aetiology classification.

Variable (mean (SD))	Healthy control (*n* = 20)	IGT (*n* = 18)	Type 2 diabetes (*n* = 20)	Idiopathic (*n* = 43)	Other (*n* = 8)	IGT vs. type 2 diabetes	*p*‐value (ANOVA)
Sex, (Male)	10 (40%)	7 (38.9%)	10 (50%)	17 (39.53)	4 (50%)		
Age (years)	41.3 (17.3)	59.11 (11.64)	59.75 (9.45)	54.72 (12.25)	62.75 (5.95)	0.948	.139
HbA1C (mean (SD)	5.5 (0.4)	5.88 (0.35)	6.97 (0.92)	5.43 (0.28)	5.58 (0.13)	**<0.001**	**<.001**
RODS	NaN (NA)	43.94 (8.41)	40.21 (6.76)	40.58 (6.83)	44.67 (5.43)	0.191	.264
ONLS	NaN (NA)	0.65 (0.79)	1.79 (1.89)	1.13 (1.12)	0.67 (0.82)	**0.031**	.070
mTCNS	NaN (NA)	9.64 (5.63)	11.08 (8.67)	13.00 (6.66)	10.50 (5.00)	0.643	.559
TCNS	NaN (NA)	6.59 (3.08)	8.10 (4.66)	7.92 (4.26)	8.00 (2.14)	0.343	.646
CNFL (mm/mm^2^)	12.53 (2.87)	4.91 (1.70)	5.49 (1.97)	4.59 (1.83)	4.52 (1.64)	0.206	.310
DCDM (cells/mm^2^)	1.47 (1.90)	13.28 (9.86)	7.81 (6.09)	8.07 (8.19)	8.35 (10.47)	**0.045**	.136
DCDI (cells/mm^2^)	2.17 (3.35)	13.69 (12.86)	11.25 (14.13)	10.06 (12.37)	7.46 (7.74)	0.496	.641
DCDT (cells/mm^2^)	1.82 (2.16)	13.49 (9.80)	9.53 (8.83)	9.06 (9.15)	7.91 (8.66)	0.168	.325

Abbreviations: CNFL, Corneal Nerve Fibre Length; DCDI, Dendritic Cell Density Immature; DCDM, Dendritic Cell Density Mature; DCDT, Dendritic Cell Density Total; HbA1C, Haemoglobin A1C; LDI, Laser Doppler Imaging; MTCNS, Modified Toronto Clinical Neuropathy Score; ONLS, Overall Neuropathy Limitations Scale; RODS, Rasch‐built Overall Disability Scale; TCNS, Toronto Clinical Neuropathy Score.

There was no significant difference in mean DCD differences across the mild, moderate and severe neuropathy severity groups, as indicated by *p*‐values of .48, .19 and .19 for each comparison.

## 4 DISCUSSION

The main finding of our study is that patients with SFN and glucose dysregulation exhibited a significant increase in corneal DCD compared with healthy controls. Additionally, other acquired types of SFN also demonstrated elevated corneal DCD. Importantly, there was no difference in DCD between SFN patients with and without glucose dysregulation. Notably, patients with IGT have higher levels of mature DCs than those with type 2 diabetes and healthy individuals.

Our study demonstrated that increased DCD is a common phenomenon across various acquired causes of SFN and is significantly higher compared with healthy controls. This finding aligns with the idea that the presence of DCD is an immune response to nerve damage, independent of its cause. Previous studies have also demonstrated the presence of DCD in infections and immune neuropathies.^25^, ^26^

We described elevated DCD in patients who met diagnostic criteria for SFN and glucose dysregulation. Our findings are consistent with other studies in diabetic patients without confirmed neuropathy, which also showed increased corneal DCD compared with healthy controls.^9^, ^10^, ^11^ Our finding of increased DCD in IGT and SFN contrasts with a study on pre‐diabetes that did not show an increase in DCD.^27^ This discrepancy may be because we defined IGT status using a 2‐hour GTT, whereas the previous study based its subject grouping on HbA1c testing, which is less sensitive.

We did not find a correlation between the clinical severity scoring (TCNS, mTCNS, ONLS, RODS) and DCD. However, there was a weak positive correlation between glycaemic control (HbA1c) and DCD. This lack of correlation between neuropathy measures and DC parameters might suggest that DCs are an epiphenomenon related to early nerve regeneration rather than nerve damage. Alternatively, the number of patients may not have allowed the discovery of any relationship. Future studies with larger cohorts are recommended to explore these relationships further.

We hypothesize that corneal DCs may play a role in nerve repair during the early stages of dysglycaemia, such as IGT, by modulating neuroinflammation. In this phase, an increased presence of mature DCs likely supports nerve repair mechanisms.^28^ However, as neuronal damage progresses, a reduction in DC numbers may impair the corneal nerve repair process, potentially exacerbating neuropathy due to diminished regenerative capacity, as observed in animal models.^29^

Our study has limitations, including sample size and measurement error. Elevated DCD can be influenced by other factors, such as allergic conjunctivitis, keratitis, dry eyes and contact lens wear.^28^, ^30^, ^31^ As our study is retrospective, we cannot account for these potential confounding factors, which should be considered in future research.

Future research should focus on large‐scale retrospective, observational and prospective studies to precisely characterize corneal DCs in humans, examining their density, distribution and correlation with nerve damage across various stages of dysglycaemia (IGT, early diabetes, advanced diabetes). These studies should also account for other co‐variables that may impact outcomes, including medication use,^32^ aging^33^ and kidney function.^34^ Collectively, these factors would provide crucial insights into the specific role of DCs in SFN. Subsequently, clinical trials could investigate the potential of using corneal DCs as biomarkers to identify patients who may benefit from disease‐modifying therapies aimed at enhancing nerve repair.^35^

In conclusion, elevated DCD potentially plays a significant role in IGT and early stages of neuropathy, serving as a potential biomarker for early neuropathic phases with neuroprotective or therapy‐responsive implications. Our data indicate that higher mature DCD levels are significant in SFN with glucose dysregulation, suggesting a common immune‐mediated mechanism for neuropathy in these individuals.

## CONFLICT OF INTEREST STATEMENT

Juan Francisco Idiaquez Rios has no conflicts of interest to declare. Carolina Barnett has received honoraria for consulting and/or advisory boards for Sanofi, Alexion and Argenx. She has received research grants from Grifols and Octapharma, not related to this work. BAP has received honoraria for educational events from Medtronic, Novo Nordisk, Sanofi, Insulet and Abbott. His research institute has received funding from BMO Bank of Montreal and Novo Nordisk for research support. He has served as an advisor to Boehringer Ingelheim, Sanofi, Insulet, Abbott, Nephris and Vertex. Vera Bril has received fees for consultancy from Grifols, CSL, UCB, Argenx, Takeda, Alnylam Octapharma, Pfizer, Powell Mansfield Inc., Akcea, Ionis Immunovant, Sanofi, Momenta (J&J), Roche, Janssen, AZ‐Alexion, NovoNordisk and research support: AZ‐Alexion, Grifols, CSL, UCB, Argenx, Takeda, Octapharma, Akcea, Momenta (J&J), Immunovant, Ionis. No funding was received for the publication of this article.

## Data Availability

The data that support the findings of this study are available on request from the corresponding author. The data are not publicly available due to privacy or ethical restrictions.

## References

[1] Sumner CJ , Sheth S , Griffin JW , Cornblath DR , Polydefkis M . The spectrum of neuropathy in diabetes and impaired glucose tolerance. Neurology. 2003;60(1):108‐111. doi:10.1212/wnl.60.1.108 12525727

[2] Asghar O , Petropoulos IN , Alam U , et al. Corneal confocal microscopy detects neuropathy in subjects with impaired glucose tolerance. Diabetes Care. 2014;37(9):2643‐2646. doi:10.2337/dc14-0279 24969581 PMC4140158

[3] Ziegler D , Papanas N , Zhivov A , et al. Early detection of nerve fiber loss by corneal confocal microscopy and skin biopsy in recently diagnosed type 2 diabetes. Diabetes. 2014;63(7):2454‐2463. doi:10.2337/db13-1819 24574045

[4] Mokhtar SBA , van der Heide FCT , Oyaert KAM , et al. (pre)diabetes and a higher level of glycaemic measures are continuously associated with corneal neurodegeneration assessed by corneal confocal microscopy: the Maastricht study. Diabetologia. 2023;66(11):2030‐2041. doi:10.1007/s00125-023-05986-5 37589735 PMC10541833

[5] Gad H , Petropoulos IN , Khan A , et al. Corneal confocal microscopy for the diagnosis of diabetic peripheral neuropathy: a systematic review and meta‐analysis. J Diabetes Investig. 2022;13(1):134‐147. doi:10.1111/jdi.13643 PMC8756328 34351711

[6] Alam U , Jeziorska M , Petropoulos IN , et al. Diagnostic utility of corneal confocal microscopy and intra‐epidermal nerve fibre density in diabetic neuropathy. PLoS One. 2017;12(7):e0180175. doi:10.1371/journal.pone.0180175 28719619 PMC5515394

[7] Pritchard N , Edwards K , Russell AW , Perkins BA , Malik RA , Efron N . Corneal confocal microscopy predicts 4‐year incident peripheral neuropathy in type 1 diabetes. Diabetes Care. 2015;38(4):671‐675. doi:10.2337/dc14-2114 25573881

[8] Leppin K , Behrendt AK , Reichard M , et al. Diabetes mellitus leads to accumulation of dendritic cells and nerve fiber damage of the subbasal nerve plexus in the cornea. Invest Ophthalmol vis Sci. 2014;55(6):3603‐3615. doi:10.1167/iovs.14-14307 24781935

[9] Colorado LH , Beecher L , Pritchard N , et al. Corneal dendritic cell dynamics are associated with clinical factors in type 1 diabetes. J Clin Med Res. 2022;11(9):2611. doi:10.3390/jcm11092611 PMC9101330 35566743

[10] Tavakoli M , Boulton AJM , Efron N , Malik RA . Increased Langerhan cell density and corneal nerve damage in diabetic patients: role of immune mechanisms in human diabetic neuropathy. Cont Lens Anterior Eye. 2011;34(1):7‐11. doi:10.1016/j.clae.2010.08.007 20851037 PMC3017662

[11] D'Onofrio L , Kalteniece A , Ferdousi M , et al. Small nerve fiber damage and Langerhans cells in type 1 and type 2 diabetes and LADA measured by corneal confocal microscopy. Invest Ophthalmol vis Sci. 2021;62(6):5. doi:10.1167/iovs.62.6.5 PMC8107645 33944892

[12] Casanova‐Molla J , Morales M , Planas‐Rigol E , et al. Epidermal Langerhans cells in small fiber neuropathies. Pain. 2012;153(5):982‐989. doi:10.1016/j.pain.2012.01.021 22361736

[13] Blanco T , Jamali A , Lopez MJ , Moein HR , Hamrah P . Plasmacytoid dendritic cells are essential for nerve repair in a mouse model of corneal injury. Invest Ophthalmol vis Sci. 2018;59(9):3327. Accessed August 6, 2024. https://iovs.arvojournals.org/article.aspx?articleid=2691221

[14] Strom A , Brüggemann J , Ziegler I , et al. Pronounced reduction of cutaneous Langerhans cell density in recently diagnosed type 2 diabetes. Diabetes. 2014;63(3):1148‐1153. doi:10.2337/db13-1444 24319115

[15] Bucher F , Schneider C , Blau T , et al. Small‐fiber neuropathy is associated with corneal nerve and dendritic cell alterations: an in vivo confocal microscopy study. Cornea. 2015;34(9):1114‐1119. doi:10.1097/ICO.0000000000000535 26186372

[16] Abraham A , Alabdali M , Alsulaiman A , et al. Laser Doppler flare imaging and quantitative thermal thresholds testing performance in small and mixed fiber neuropathies. PLoS One. 2016;11(11):e0165731. doi:10.1371/journal.pone.0165731 27824912 PMC5100917

[17] American Diabetes Association . 2. Classification and diagnosis of diabetes: standards of medical Care in Diabetes‐2021. Diabetes Care. 2021;44(Suppl 1):S15‐S33. doi:10.2337/dc21-S002 33298413

[18] Bril V , Perkins BA . Validation of the Toronto clinical scoring system for diabetic polyneuropathy. Diabetes Care. 2002;25(11):2048‐2052. doi:10.2337/diacare.25.11.2048 12401755

[19] Bril V , Tomioka S , Buchanan RA , Perkins BA , the mTCNS Study Group . Reliability and validity of the modified Toronto clinical neuropathy score in diabetic sensorimotor polyneuropathy. Diabet Med. 2009;26(3):240‐246. doi:10.1111/j.1464-5491.2009.02667.x 19317818 PMC2871179

[20] Graham RC , Hughes RAC . A modified peripheral neuropathy scale: the overall neuropathy limitations scale. J Neurol Neurosurg Psychiatry. 2006;77(8):973‐976. doi:10.1136/jnnp.2005.081547 16574730 PMC2077620

[21] van Nes SI , Vanhoutte EK , van Doorn PA , et al. Rasch‐built overall disability scale (R‐ODS) for immune‐mediated peripheral neuropathies. Neurology. 2011;76(4):337‐345. doi:10.1212/WNL.0b013e318208824b 21263135

[22] Ostrovski I , Lovblom LE , Farooqi MA , et al. Reproducibility of in vivo corneal confocal microscopy using an automated analysis program for detection of diabetic sensorimotor polyneuropathy. PLoS One. 2015;10(11):e0142309. doi:10.1371/journal.pone.0142309 26539984 PMC4634969

[23] Zhivov A , Stave J , Vollmar B , Guthoff R . In vivo confocal microscopic evaluation of Langerhans cell density and distribution in the normal human corneal epithelium. Graefes Arch Clin Exp Ophthalmol. 2005;243(10):1056‐1061. doi:10.1007/s00417-004-1075-8 15856272

[24] Idiaquez JF , Martinez R , Barnett‐Tapia C , Perkins BA , Bril V . Reliability of confocal corneal microscopy for measurement of dendritic cell density in suspected small fiber neuropathy. Muscle Nerve. 2023;68(4):460‐463. doi:10.1002/mus.27948 37534704

[25] Stettner M , Hinrichs L , Guthoff R , et al. Corneal confocal microscopy in chronic inflammatory demyelinating polyneuropathy. Ann Clin Transl Neurol. 2016;3(2):88‐100. doi:10.1002/acn3.275 26900579 PMC4748316

[26] Kemp HI , Petropoulos IN , Rice ASC , et al. Use of corneal confocal microscopy to evaluate small nerve fibers in patients with human immunodeficiency virus. JAMA Ophthalmol. 2017;135(7):795‐800. doi:10.1001/jamaophthalmol.2017.1703 28594979 PMC6583030

[27] Chao C , Wang R , Jones M , et al. The relationship between corneal nerve density and hemoglobin A1c in patients with prediabetes and type 2 diabetes. Invest Ophthalmol vis Sci. 2020;61(12):26. doi:10.1167/iovs.61.12.26 PMC7594598 33112943

[28] Wu M , Hill LJ , Downie LE , Chinnery HR . Neuroimmune crosstalk in the cornea: the role of immune cells in corneal nerve maintenance during homeostasis and inflammation. Prog Retin Eye Res. 2022;91(101105):101105. doi:10.1016/j.preteyeres.2022.101105 35868985

[29] Gao N , Lee P , Yu FS . Intraepithelial dendritic cells and sensory nerves are structurally associated and functional interdependent in the cornea. Sci Rep. 2016;6:36414. doi:10.1038/srep36414 27805041 PMC5090364

[30] Mobeen R , Stapleton F , Chao C , Madigan MC , Briggs N , Golebiowski B . Corneal epithelial dendritic cell density in the healthy human cornea: a meta‐analysis of in‐vivo confocal microscopy data. Ocul Surf. 2019;17(4):753‐762. doi:10.1016/j.jtos.2019.07.001 31279064

[31] Tajbakhsh Z , Golebiowski B , Stapleton F , et al. Increased dendritic cell density and altered morphology in allergic conjunctivitis. Eye. 2023;37(14):2896‐2904. doi:10.1038/s41433-023-02426-x 36747109 PMC10516863

[32] Asiedu K , Tummanapalli SS , Alotaibi S , et al. Impact of SGLT2 inhibitors on corneal nerve morphology and dendritic cell density in type 2 diabetes. Ocul Immunol Inflamm. 2024;32(2):234‐241. doi:10.1080/09273948.2023.2263789 37801679

[33] Sharma S , Tobin V , Vas PRJ , Malik RA , Rayman G . The influence of age, anthropometric and metabolic variables on LDIFLARE and corneal confocal microscopy in healthy individuals. PLoS One. 2018;13(3):e0193452. doi:10.1371/journal.pone.0193452 29518115 PMC5843248

[34] Asiedu K , Markoulli M , Tummanapalli SS , et al. Impact of chronic kidney disease on corneal neuroimmune features in type 2 diabetes. J Clin Med. 2022;12(1):16. doi:10.3390/jcm12010016 36614815 PMC9820846

[35] Ponirakis G , Al‐Janahi I , Elgassim E , et al. Glucose‐lowering medication associated with weight loss may limit the progression of diabetic neuropathy in type 2 diabetes. J Peripher Nerv Syst. 2024;29(4):406‐414. doi:10.1111/jns.12664 PMC11625975 39439079

